# Effect of Delta-9-Tetrahydrocannabinol on Mouse Resistance to Systemic *Candida albicans* Infection

**DOI:** 10.1371/journal.pone.0103288

**Published:** 2014-07-24

**Authors:** Gideon W. Blumstein, Arya Parsa, Anthony K. Park, Beverly L. P. McDowell, Melissa Arroyo-Mendoza, Marie Girguis, Jill P. Adler-Moore, Jon Olson, Nancy E. Buckley

**Affiliations:** Department of Biological Sciences, California State Polytechnic University, Pomona, California, United States of America; Institute of Microbiology, Switzerland

## Abstract

Delta-9-tetrahydrocannabinol (Δ^9^-THC), the psychoactive component of marijuana, is known to suppress the immune responses to bacterial, viral and protozoan infections, but its effects on fungal infections have not been studied. Therefore, we investigated the effects of chronic Δ^9^-THC treatment on mouse resistance to systemic *Candida albicans* (*C. albicans*) infection. To determine the outcome of chronic Δ^9^-THC treatment on primary, acute systemic candidiasis, c57BL/6 mice were given vehicle or Δ^9^-THC (16 mg/kg) in vehicle on days 1–4, 8–11 and 15–18. On day 19, mice were infected with 5×10^5^ *C. albicans*. We also determined the effect of chronic Δ^9^-THC (4–64 mg/kg) treatment on mice infected with a non-lethal dose of 7.5×10^4^ *C. albicans* on day 2, followed by a higher challenge with 5×10^5^ *C. albicans* on day 19. Mouse resistance to the infection was assessed by survival and tissue fungal load. Serum cytokine levels were determine to evaluate the immune responses. In the acute infection, chronic Δ^9^-THC treatment had no effect on mouse survival or tissue fungal load when compared to vehicle treated mice. However, Δ^9^-THC significantly suppressed IL-12p70 and IL-12p40 as well as marginally suppressed IL-17 versus vehicle treated mice. In comparison, when mice were given a secondary yeast infection, Δ^9^-THC significantly decreased survival, increased tissue fungal burden and suppressed serum IFN-γ and IL-12p40 levels compared to vehicle treated mice. The data showed that chronic Δ^9^-THC treatment decreased the efficacy of the memory immune response to candida infection, which correlated with a decrease in IFN-γ that was only observed after the secondary candida challenge.

## Introduction

Candidiasis refers to a fungal infection caused by yeast of the genus candida, predominantly *Candida albicans* (*C. albicans*) [1], [2]. Although *C. albicans* is part of the normal microbiota, when it overgrows at a given site in the body, it can lead to serious infections. The two major types of candidiasis are mucocutaneous candidiasis and systemic candidiasis. Mucocutaneous candidiasis is caused by overgrowth of candida on mucosal surfaces such as respiratory, gastrointestinal and reproductive systems [3], [4]. In immune compromised individuals, mucosal candidiasis can be associated with an increased incidence of systemic candidiasis [5], [6]. Systemic candidiasis is often life-threatening and results from the presence of the yeast in the bloodstream and subsequent spread of the yeast into the organs [4]. Candida species are the fourth leading cause of nosocomial bloodstream infections in the U.S [7].

Host resistance against the spread of candida throughout the body requires a complex immune response which involves both the innate and the adaptive immune systems [8], [9]. Extracellular and intracellular pattern recognition receptors on cells of the innate immune system recognize different components of *C. albicans*. Phagocytic cells, predominantly neutrophils, which have these receptors, are largely responsible for clearing *C. albicans* infections. In addition, ingestion and presentation of the yeast by dendritic cells, professional antigen presenting cells, induce Th1 and Th17 immunity which is responsible for the resistance and development of protective immunity against *C. albicans* [8]–[11].

Delta-9-tetrahydrocannabinol (Δ^9^-THC), the psychoactive component of marijuana, has been widely reported to alter immune responses. Δ^9^-THC is known to modulate cytokine production, alter splenocyte proliferation, suppress antibody production, impair macrophage antigen presentation and inhibit chemotaxis [12]. However, despite these effects, there are only a limited number of studies on the effects of Δ^9^-THC on host resistance to infection. This is an area that needs to be further investigated since cannabis is used regularly by many patients to provide symptomatic relief for a broad range of conditions such as multiple sclerosis, chronic pain, nausea and emesis, loss of appetite and weight in cancer and AIDS patients, addiction, and metabolic disorders [13]. There are studies that have shown that Δ^9^-THC suppresses the immune response to bacterial [14]–[16], viral [17] and protozoan [18] infections, but nothing is known about the effects of Δ^9^-THC on fungal infections. In the present study, we investigated the effects of chronic Δ^9^-THC treatment on the resistance to systemic *C. albicans* infection in mice. Specifically, we studied the effect of chronic Δ^9^-THC treatment on mouse resistance to a primary, acute systemic *C. albicans* infection as well as to a secondary systemic *C. albicans* infection. We assessed the responses to the two different types of infections by monitoring survival, measuring kidney, liver, brain and spleen fungal load and by analyzing cytokine production.

## Materials and Methods

### Animals

Female c57BL/6 mice 8–12 weeks of age were purchased from Harlan Laboratories (Indianapolis, IN). The animals were housed in the AALAC certified animal housing facility at California State Polytechnic University, Pomona. They were maintained at room temperature with a 12-hour light/dark cycle and given food and water *ad libitum*. Mice were used according to guidelines established by the California State Polytechnic University, Pomona’s Animal Care and Use Committee (ACUC). ACUC approved the procedures carried out for this project under Animal Protocol Number 13.015.

### Δ^9^-Tetrahydrocannabinol (Δ^9^-THC) preparation and animal drug treatment

Δ^9^-THC in ethanol was provided by the National Institute on Drug Abuse (NIDA, Bethesda, MD). Similar to the procedure described by others [18], [19], Δ^9^-THC in ethanol was mixed with cremophor and saline to obtain a Δ^9^-THC, cremophor, saline preparation in a 1∶1∶18 ratio. As the vehicle control, we used ethanol (EtOH; Sigma-Aldrich, St.Louis, MO), cremophor (Sigma-Aldrich, St.Louis, MO), saline (1∶1∶18). The Δ^9^-THC or vehicle control was administered via intraperitoneal (i.p.) injection. Where indicated, mice received vehicle or 4, 8, 16, 32 or 64 mg Δ^9^-THC/kg (200 µl/20 g mouse, i.p.) on days 1 through 4, 8 through 11 and 15 through 18. This dosing regimen was selected based on the procedure described by Cabral and Marciano-Cabral [18]. These Δ^9^-THC doses were comparable to those used by others in bacterial [15], [16], [20], viral [21] and parasitic [18] mouse infection models.

### *Candia albicans* preparation and yeast challenge of mice

*Candida albicans* (*C. albicans* strain CP 620) was prepared as previously described [22]. Briefly, *C. albicans* (0.5 ml) was grown in 50 ml Sabouraud's Dextrose Broth (SAB) (BD-Difco, Franklin Lakes, NJ) at 35°C for 20 hours (h). After the incubation, 0.5 ml of the *C. albicans* culture was transferred into 50 ml of SAB and incubated at room temperature (25°C) for another 20 h. After this 20 h incubation, 0.5 ml of the *C. albicans* culture was again transferred into 50 ml SAB and incubated at room temperature for 22 h. *C. albicans* was then washed 3 times with sterile 1X Phosphate Buffered Saline (PBS). The yeast cells were then pelleted and re-suspended in 1X PBS and counted using 0.5% methylene blue. The *C. albicans* cell number was adjusted with 1X PBS to the proper concentrations to be injected intravenously (i.v.) into each mouse. To investigate the effect of chronic Δ^9^-THC on primary, acute systemic *C. albicans* infection, mice were infected i.v. with 5×10^5^ *C. albicans* cells in 100 µl on day 19. To investigate the effect of chronic Δ^9^-THC C on the response to a secondary challenge with *C. albicans*, mice were first administered 7.5×10^4^ *C. albicans* cells i.v. in 100 µl on day 2 and then challenged with 5×10^5^ *C. albicans* cells i.v. in 100 µl on day 19.

Mice used for survival (n = 7/treatment) were monitored daily for up to 2 weeks after the last yeast infection. They were observed for symptoms of distress such as weight loss (>20%), ability to get up and down, activity level, and hair coat appearance. To minimize distress, each mouse was scored daily for each distress symptom where 0 indicated that the mouse was in good condition and 3 indicated that the mouse exhibited the worst score for that symptom. If the mouse had a score of 3 for any symptom or the sum of the highest 3 scores was over 6, the mouse was euthanized by CO_2_ inhalation followed by cervical dislocation. Because this work focused on investigating the effects of Δ^9^-THC on mouse resistance to systemic *C. albicans* infection, the fungal infection must be measurable (i.e. weight loss, mortality and activity level). Any intervention to alleviate pain (other than euthanasia) would have obfuscated the results by altering animal behavior and changing observable symptoms. Therefore, it was required that upon yeast infection, the experiments be carried out without the use of anesthetics or analgesics.

### Tissue collection and assessment of survival

To assess tissue fungal load in the kidneys, spleen, brain and liver and serum cytokine levels, mice (n = 4–7 mice per treatment group) were anesthetized with 16 mg/kg of xylazine, 80 mg/kg of ketamine in saline (200 µl/20 g mouse, i.p.) at 4–24 h or 14 days after the last yeast challenge. Mice that were sacrificed 14 days after the last yeast infection were monitored daily as described above. The mice were exsanguinated via cardiac puncture, and the blood centrifuged to obtain the serum used for cytokine analysis (described below). The kidneys, spleen, brain and liver were collected to assess tissue fungal load as determined by the formation of the *C. albicans* colony forming units (described below). To determine the effect of Δ^9^-THC on survival, mice (n = 7 per treatment group) were observed for morbidity and mortality for up to 2 weeks after the 5×10^5^ *C. albicans* cell challenge.

### Colony Forming Units (CFU) Assay

Tissue (kidneys, spleen, brain, liver) fungal burden was determined for each mouse as previously described [22]. Briefly, tissues were homogenized in 1 ml of PBS containing 0.05% chloramphenicol (Sigma-Aldrich, St. Louis, MO). The tissue homogenate was then diluted (1∶50–1∶500) in PBS containing 0.05% chloramphenicol, and 200 µl of each dilution was plated onto SAB agar plates containing 0.05% chloramphenicol. The plates were incubated for 24–48 h at 37°C after which the *C. albicans* colonies were counted. The data is expressed as the Log of colony forming units (Log_10_ CFU) per gram of tissue.

### Cytokine analysis

To determine the serum levels of multiple cytokines (interleukin-1α (IL-1α), IL-1β, IL-4, IL-6, IL-10, IL-12p70, IL-17, interferon-γ (IFN-γ) and tumor necrosis factor-α (TNF-α)), we utilized the Bio-Plex Bead Assay (Bio-Rad, Hercules, CA) according to the manufacturer’s instructions using the Luminex100 plate reader (Luminex Corp., Austin, TX). In addition, IFN-γ and IL-12p40 serum levels were further analyzed using cytokine specific enzyme-linked immunosorbent assay kits (BD Biosciences, San Diego, CA) following the manufacturer’s instructions.

### Statistical analysis

Statistical analysis was performed using GraphPad Prism Version 6.0. Statistical significance of survival studies was done using the Log-rank (Mantel-Cox) test. Statistical significance of tissue fungal load and cytokine levels was obtained by carrying out one-way analysis of variance (ANOVA) followed by Tukey’s test to compare all treatments within a given time point. One-way ANOVA followed by Dunnett’s test was used to compare all treatments against values obtained for the earliest time point vehicle or Δ^9^-THC treatment. In addition, statistical significance of serum cytokine levels between vehicle and Δ^9^-THC at 16 mg/kg was determined by unpaired parametric two-tailed t-test. The statistical analysis used is specified in each Figure legend. In all cases, p<0.05 was considered statistically significant.

## Results

In this study, we investigated the effects of chronic Δ^9^-THC treatment on mouse resistance to primary, acute systemic *C. albicans* infection as well as secondary systemic *C. albicans* infection using survival and tissue fungal load as the endpoints. We also examined the immune responses in these mice by measuring the serum cytokine levels.

### Effect of chronic Δ^9^-THC treatment on the response of mice to acute systemic *C. albicans* challenge

**Figure 1:**
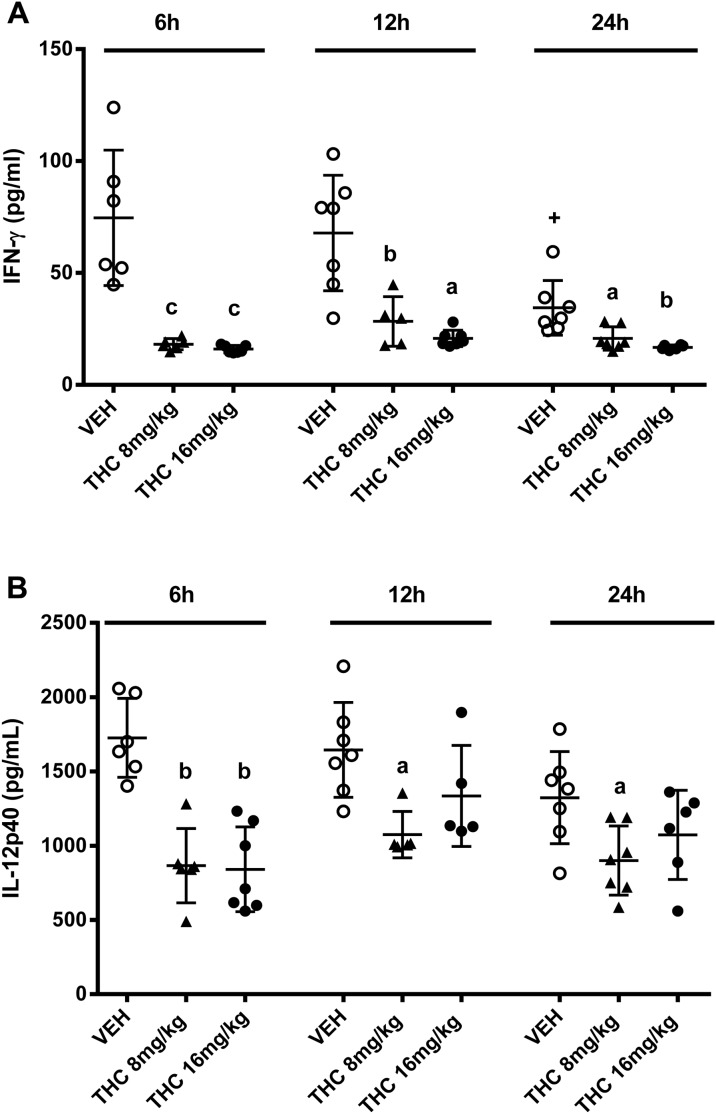
Δ^9^-THC treatment did not alter survival of mice acutely infected with *C. albicans*. Female (F) c57BL/6 mice (n = 7/group) received vehicle or Δ^9^-THC (16 mg/kg) on days 1–4, 8–11 and 15–18. The mice were then infected with *C. albicans* (5×10^5^ yeast cells/mouse(ms), i.v.) on day 19. The mice were monitored daily for survival. The data is representative of two experiments.

To determine the effect of chronic Δ^9^-THC treatment on the resistance to a primary, acute systemic *C. albicans* infection, mice were treated with vehicle or Δ^9^-THC (16 mg/kg) on days 1–4, 8–11 and 15–18 and challenged with *C. albicans* (5×10^5^ yeast cells/mouse, i.v.) on day 19. Survival of mice given vehicle was 43% and that for mice given the chronic Δ^9^-THC treatment was 57%, but these were not statistically different (Fig. 1). This demonstrates that chronic Δ^9^-THC treatment did not significantly alter mouse resistance to this type of *C. albicans* infection.

In addition to survival, another important indicator of disseminated candidiasis is tissue fungal invasion, and the severity of the infection can be assessed by measuring tissue fungal load based on yeast colony forming units/g tissue (CFU). One of the organs known to have the highest level of yeast burden in a systemic *C. albicans* infection is the kidney [23], [24]. Thus, we determined the number of *C. albicans* CFUs in kidneys from infected mice treated with vehicle or Δ^9^-THC. We found that tissue fungal load increased similarly in both vehicle and Δ^9^-THC treated animals over the course of infection throughout the 14 days of the study (Table 1), suggesting again that chronic Δ^9^-THC did not significantly alter mouse resistance to this type of *C. albicans* infection.

**Table 1:** Chronic Δ^9^-THC treatment does not alter kidney fungal load (Log_10_ CFU/g) compared to Vehicle in mice acutely infected with *C. albicans.*

Treatment	6 h	12 h	24 h	14 days
**Vehicle**	4.71±0.25	4.97±0.09	5.73±0.47^a^	5.89±0.86^b^
**Δ^9^-THC 16 mg/kg**	4.92±0.05	5.16±0.26	5.95±0.39^c^	6.33±0.74^d^

Female c57BL/6 mice (n = 5–7/treatment) were treated with vehicle or Δ^9^-THC at 16 mg/kg on days 1–4, 8–11 and 15–18. On day 2, mice were infected with 5×10^5^ *C. albicans*/mouse. Colony forming units/g (Log_10_ CFU/g) are shown for kidneys collected 6 h, 12 h, 24 h or 14 days after the yeast infection. ^a^p = 0.04 compared to vehicle treatment at 6 h; ^b^p = 0.01 compared to vehicle treatment at 6 h; ^c^p = 0.009 compared to Δ^9^-THC 6 h; ^d^p = 0.0003 compared to Δ^9^-THC 6 h. Statistics determined by one way ANOVA followed by Dunnett’s test.

**Figure 2:**
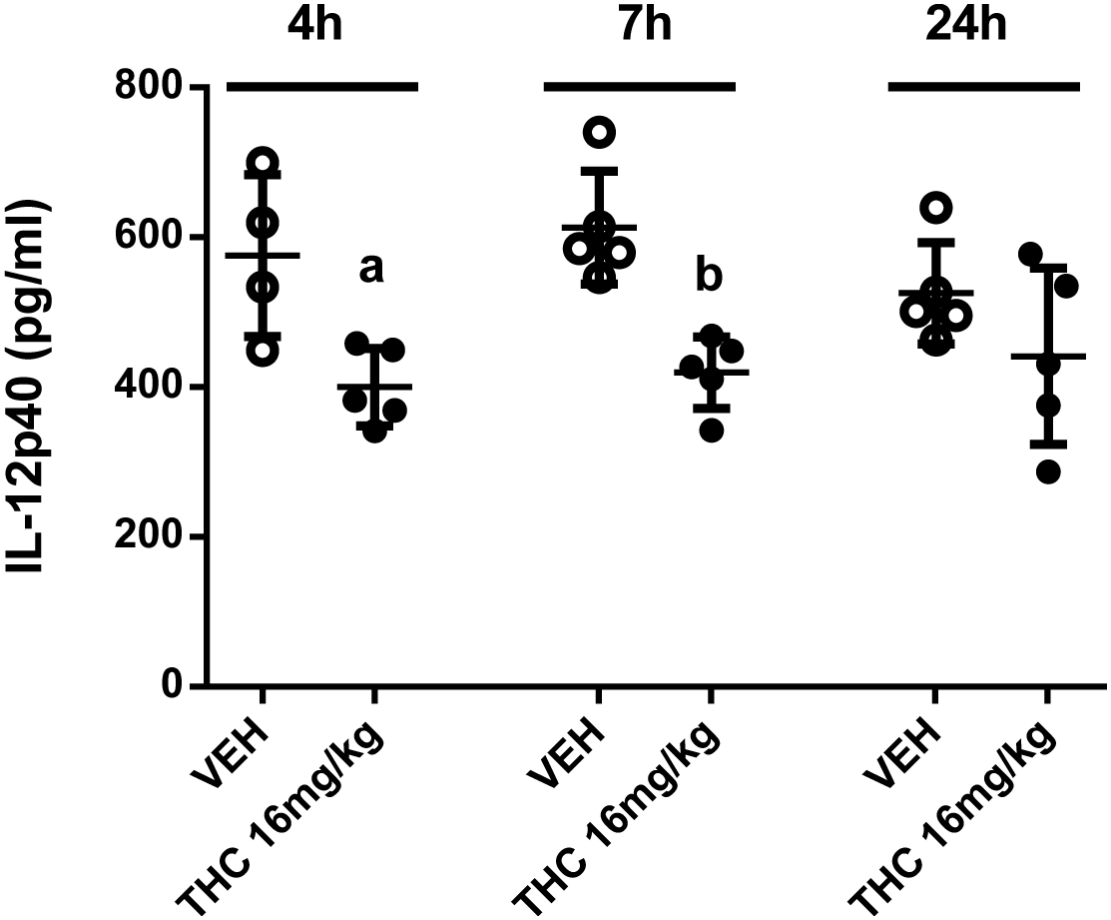
Chronic Δ^9^-THC treatment decreases serum IL-12p40 in mice acutely infected with *C. albicans*. Female c57BL/6 mice (n = 4–5/group) received vehicle or Δ^9^-THC (16 mg/kg) on days 1–4, 8–11 and 15–18. Mice were then infected with *C. albicans* (5×10^5^ yeast cells/mouse, i.v.) on day 19. Serum IL-12p40 was determined 4 h, 7 h and 24 h after the yeast infection via ELISA. Compared to vehicle treated animals at corresponding time points: ^a^p = 0.014 and ^b^p = 0.0013 as analyzed by unpaired parametric two-tailed t-test.

We measured serum cytokine levels since cytokines are key mediators of the immune response and cannabinoids have been shown to modulate cytokine production [25]. Of the serum cytokines analyzed 3 days post-challenge, chronic Δ^9^-THC (16 mg/kg) treatment significantly reduced IL-12p70 (vehicle treated mice 241±35 pg/ml vs Δ^9^-THC treated mice 178±20 pg/ml, p = 0.021) and marginally decreased IL-17 (vehicle treated mice 183±39 pg/ml vs Δ^9^-THC treated mice 119±36 pg/ml, p = 0.055). We also determined the IL-12p40 levels in serum, the regulatory component of IL12p70 [26], at 4 h, 7 h and 24 h after the yeast challenge. The IL-12p40 levels were significantly decreased at 4 h and 7 h and less so at 24 h compared to vehicle treated mice (Fig. 2). These observations suggest that chronic Δ^9^-THC treatment reduced IL-12p70 serum levels by suppressing IL-12p40 production. Taken together, these data demonstrate that chronic Δ^9^-THC treatment suppressed production of some cytokines in the mice which were given a primary acute challenge with *C. albicans*, although the severity of the infection was the same as that seen with vehicle treated mice.

### Effect of chronic Δ^9^-THC treatment on the response of mice to a secondary systemic challenge with *C. albicans*

**Figure 3:**
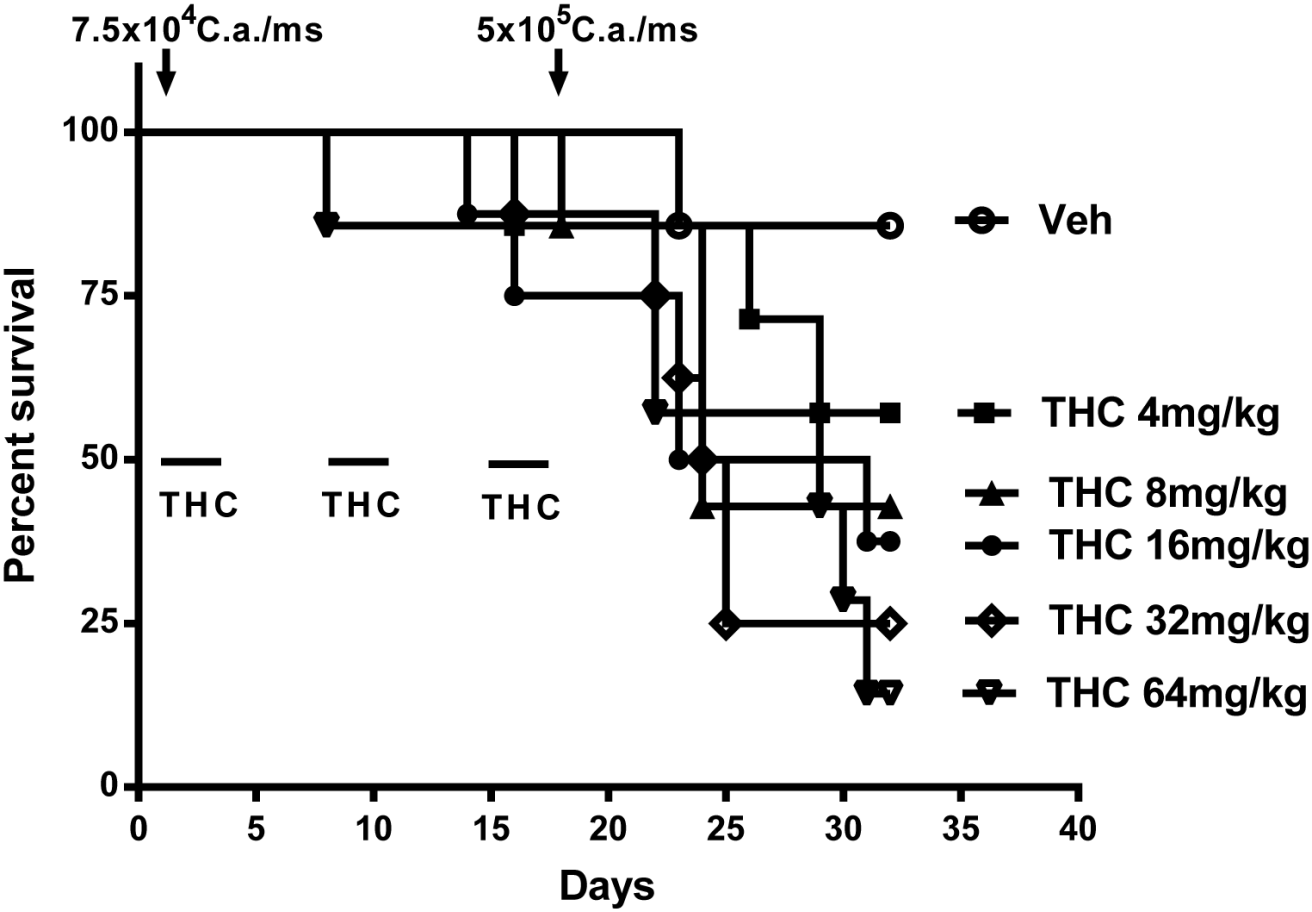
Chronic Δ^9^-THC treatment and survival of mice given a secondary systemic challenge with *C. albicans*. Female c57BL/6 mice (n = 7/group) received vehicle or Δ^9^-THC at the indicated concentrations on days 1–4, 8–11 and 15–18. The mice were challenged with *C. albicans* (7.5×10^4^ yeast cells/mouse (ms), i.v.) on day 2 and again challenged with *C. albicans* (5×10^5^ yeast cells/ms, i.v.) on day 19. The mice were monitored daily for survival. Compared to the survival of vehicle treated mice, the p values are as follows: Δ^9^-THC 4 mg/kg (p = 0.28), Δ^9^-THC 8 mg/kg (p = 0.14), Δ^9^-THC 16 mg/kg (p = 0.06), Δ^9^-THC 32 mg/kg (p = 0.03) and Δ^9^-THC 64 mg/kg (p = 0.01). The data are representative of two experiments.

To determine the effect of chronic Δ^9^-THC treatment on a secondary systemic *C. albicans* infection, mice were treated with vehicle or different concentrations of Δ^9^-THC (4, 8, 16, 32 or 64 mg/kg, i.p.) on days 1–4, 8–11 and 15–18. Mice were challenged with a non-lethal dose of 7.5×10^4^ *C. albicans* cells/mouse on day 2 and on day 19, challenged with 5×10^5^ *C. albicans* cells/mouse. Compared to vehicle treated mice, survival of mice treated with Δ^9^-THC at 4 mg/kg or 8 mg/kg was not statistically significantly different (Fig. 3). Survival of mice treated with Δ^9^-THC at 16 mg/kg was markedly decreased although not statistically significant (p>0.06). However, survival of mice given Δ^9^-THC at 32 mg/kg or 64 mg/kg was significantly decreased compared to that of vehicle treated mice (Fig. 3).

### Chronic Δ^9^-THC treatment increases tissue fungal burden in mice following a secondary systemic *C. albicans* challenge

The fungal load in the kidneys, liver, spleen and brain of mice at an early time-point (6 h post-yeast challenge) following treatment with 8 mg/kg or 16 mg/kg Δ^9^-THC was significantly increased compared to vehicle treated mice following a secondary challenge with *C. albicans* (Table 2). In both vehicle and Δ^9^-THC treated mice, kidneys and liver exhibited the largest fungal load which was 2–4 logs higher than the fungal load in the spleen and brain. The increase in tissue fungal load that accompanied the chronic Δ^9^-THC treatment showed that the Δ^9^-THC enhanced the severity of the infection.

**Table 2:** Chronic Δ^9^-THC increases tissue fungal load in mice receiving a secondary *C. albicans* infection.

Treatment	Kidney	Liver	Brain	Spleen
**Vehicle**	4.56±1.19	4.47±0.22	1.80±0.29	2.32±0.30
**Δ^9^-THC 8mg/kg**	6.69±0.48^a^	6.47±0.82^c^	2.65±0.27^d^	2.62±0.38
**Δ^9^-THC 16mg/kg**	6.30±0.97^b^	6.39±0.65^c^	3.12±0.10*^e^	2.88±0.20^f^

Female c57BL/6 mice (n = 5–7) were treated with vehicle or Δ^9^-THC 16 mg/kg on days 1–4, 8–11 and 15–18. On day 2, mice were infected with 7.5×10^4^ *C. albicans*/mouse. On day 19, mice were injected with 5×10^5^ *C. albicans*/mouse. Six hours after the second yeast injection, tissues were harvested and colony forming units (CFU) were determined for the indicated tissues. Data is expressed as Log_10_ CFU/g tissue. For kidneys: ^a^p = 0.0006, ^b^p = 0.0053 compared to vehicle control; liver: ^c^p<0.0001 compared to vehicle control; brain: ^d^p = 0.0007, ^e^p<0.0001 compared to vehicle control, *p = 0.003 compared to Δ^9^-THC 8 mg/kg treatment; spleen, ^f^p = 0.03 compared to vehicle control. Statistical tests: ANOVA followed by Tukey’s test.

### Chronic Δ^9^-THC treatment suppresses IFN-γ and IL-12p40 in mice following a secondary systemic *C. albicans* challenge

**Figure 4:**
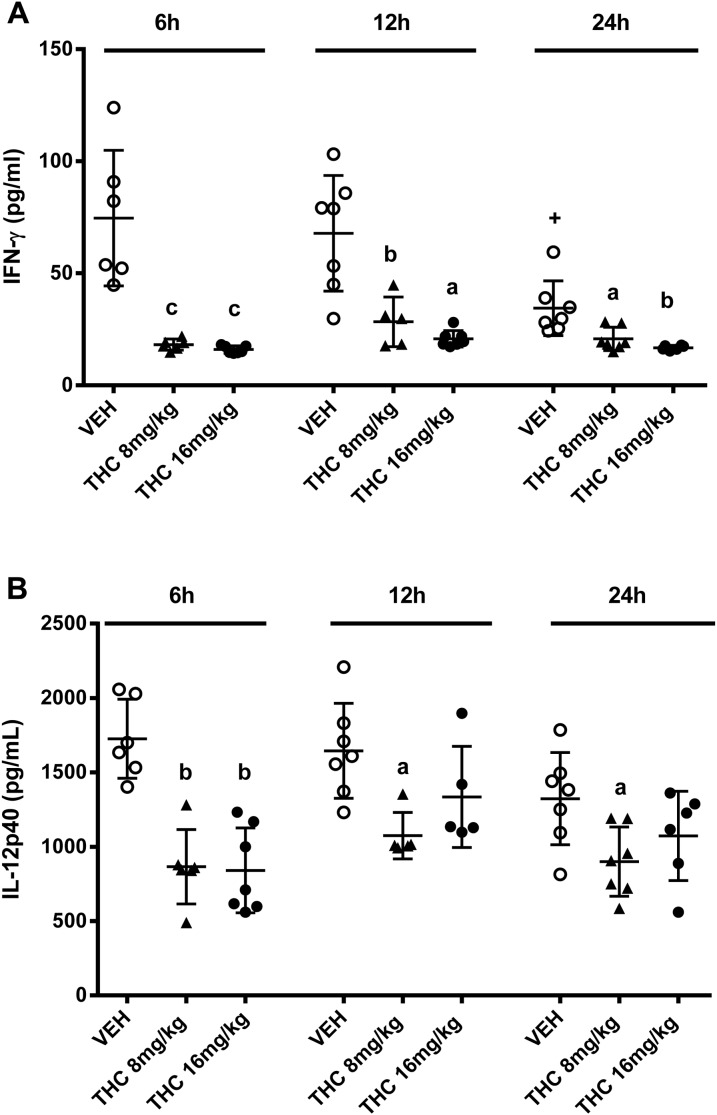
Chronic Δ^9^-THC decreases serum IFN-γ and IL-12p40 levels in the secondary systemic *C. albicans* infection. Female c57BL/6 mice (n = 5–7/group) were given vehicle or Δ^9^-THC at 8 mg/kg or 16 mg/kg on days 1–4, 8–11 and 15–18. Mice were injected with *C. albicans* (7.5×10^4^ yeast cells/ms, i.v.) on day 2 and challenged again with *C. albicans* (5×10^5^ yeast cells/ms, i.v.) on day 19. Six, twelve or twenty four hours after receiving the second *C.albicans* challenge, blood was collected for IFN-γ (A) and IL-12 (B) analysis via ELISA. For (A) ^a^p≤0.013 compared to the corresponding time point for vehicle treated mice (VEH); ^b^p≤0.003 compared to the corresponding time point for VEH; ^c^p≤0.0002 compared to the corresponding time point for VEH; Statistical analysis: one way ANOVA followed by Tukey’s multiple comparison test within time points; **^+^**p≤0.04 compared to VEH mice at 6 h and at 12 h; Statistical analysis: one way ANOVA followed by Dunnett’s. For (B) ^a^p≤0.03 compared to the corresponding time point for VEH; ^b^p≤0.0001 compared to the corresponding time point or VEH; Statistical analysis: one way ANOVA followed by Tukey’s multiple comparison test within time points. Each symbol in the Figures represents one mouse. This data is representative of two experiments.

Serum cytokine levels were assessed by ELISA at various times after the second yeast challenge. Compared to the vehicle controls, Δ^9^-THC (8 mg/kg or 16 mg/kg) reduced IFN-γ serum levels at all time-points studied (Fig. 4A). Furthermore, in this time course study, vehicle treated mice had IFN-γ serum levels which were highest at 6 h and 12 h, but substantially decreased by 24 h (Fig. 4A). While serum IL-12p70 levels were not decreased by Δ^9^-THC treatment, serum IL-12p40 levels were suppressed by Δ^9^-THC in this infection. Serum obtained 6 h after the second yeast challenge revealed that Δ^9^-THC at both 8 mg/kg and 16 mg/kg significantly decreased IL-12p40 serum levels compared to levels seen in vehicle control mice (Fig. 4B). IL-12p40 serum levels 12 h and 24 h after the second yeast challenge were also significantly decreased by Δ^9^-THC at 8 mg/kg, but less so with Δ^9^-THC at 16 mg/kg.

The data show that although chronic Δ^9^-THC treatment decreased the production of these two cytokines, the increased severity of the Candida infection in Δ^9^-THC treated mice given a second challenge with *C. albicans* could only be correlated with decreased IFN-γ production, since serum IL-12p40 serum levels were also significantly decreased in the mice given a primary, acute infection with *C. albicans* and treated with Δ^9^-THC.

## Discussion

To our knowledge, this is the first reported study on the effects of Δ^9^-THC on host resistance to yeast infection. We have shown that, in a primary, acute, systemic *C. albicans* infection, chronic Δ^9^-THC treatment suppressed IL-12p70 and IL-12p40 serum levels, suggesting that chronic Δ^9^-THC treatment suppresses some of the immunity to this type of yeast infection. However, the cytokine suppression was not sufficient to decrease mouse resistance to the infection when assessed by mouse survival and tissue fungal burden. In comparison, chronic Δ^9^-THC treatment decreased mouse survival, increased tissue fungal load and suppressed serum IL-12p40 and IFN-γ when mice were given a non-lethal challenge with *C. albicans* and 17 days later given a high challenge dose with this same yeast. These data demonstrate that, in immune competent mice, there was a correlation between a significantly impaired memory immune response and chronic Δ^9^-THC treatment which enhanced the severity of infection. This was not the case in the primary immune response to systemic *C. albicans* infection.

IL-12 is produced predominantly by macrophages and dendritic cells [27]. However, suppression of this cytokine in our studies did not reduce mouse resistance to the primary acute yeast infection. Our findings are supported by work from others who have shown that IL-12 is not essential in the host response to systemic *C. albicans* infection. These investigators have found that survival and tissue fungal load of IL-12 knockout mice systemically infected with *C. albicans* was comparable to that of infected wild type mice [28], [29].

While there is controversy as to the contribution of humoral immunity to host defense against *C. albicans* infection, cell mediated immunity has been shown to be necessary [30], [31]. CD4+ T cells are essential in the cell mediated adaptive immune responses to intracellular and extracellular pathogens. When a naïve T cell recognizes an antigen-major histocompatibility complex (MHC) on an antigen presenting cell (APC), the T cell becomes activated initiating a primary response, with subsequent APC secretion of IL-12, and the conversion of naïve CD4+T cells to Th1 effectors. Th1 effectors secrete cytokines such as IFN-γ and TNF-α and some of them can differentiate into memory T cells [32].

Memory T cells are antigen-activated T cells that are highly reactive to a subsequent challenge with the same antigen, generating a secondary response, and they can become activated by macrophages, dendritic cells and B cells. Naïve T cells can only be activated by dendritic cells [33]. IFN-γ is important in sustaining this Th1 response and in stimulating phagocyte anti-fungal activity [8]. Notably, serum IFN-γ was significantly decreased in our studies in the mice that were given a secondary challenge with *C. albicans* suggesting that the decrease in this cytokine may have been critical in reducing the memory immune response in our mice. It is widely recognized that Δ^9^-THC suppresses the Th1 response to bacterial infections [14], [16] and that this suppression is caused by a reduction in IL-12p70, IL-12p40 and IFN-γ [15], [34].

We conclude that chronic Δ^9^-THC treatment significantly compromises mouse resistance to a secondary, but not to a primary, acute, systemic *C. albicans* infection in immune competent mice. Future studies need to be done to elucidate the effect of chronic Δ^9^-THC treatment on the memory immune response to this yeast, including collecting serum samples for cytokine analysis at later time-points post infection, expanding the number of cytokines to be examined, and comparing the antigen specific responses of effector T cells and memory T cells. Cannabinoid receptor expression levels also need to be investigated, as well as the effect cannabinoid receptor antagonists will have on chronic Δ^9^-THC dosing in this infection model. The effect of chronic Δ^9^-THC exposure in immune compromised animals also remains to be investigated, especially since many immune compromised individuals use cannabinoids therapeutically.
